# Brief Episodes, Lasting Impact: A Case Series on Acute and Transient Psychotic Disorder

**DOI:** 10.1192/j.eurpsy.2025.2235

**Published:** 2025-08-26

**Authors:** C. Martins, M. Sant’Ovaia, J. M. de Castro, S. Martins, L. A. Fernandes

**Affiliations:** 1 Psychiatry, Unidade Local de Saúde Amadora/Sintra, Amadora, Portugal

## Abstract

**Introduction:**

Psychotic disorders with acute onset and remitting course have been described by 19th and 20th-century European psychiatrists under various terms, such as “amentia,” “cycloid psychosis,” “bouffée délirante”. In modern taxonomy, brief psychotic episodes are classified as “acute and transient psychotic disorder” (ATPD) in ICD-11 and “brief psychotic disorder” in DSM-5. The lack of continuity between earlier nosological concepts and current descriptive categories, along with frequent changes in definitions across DSM and ICD versions, has hindered empirical research, limiting our understanding of these conditions. As a result, ATPDs have been marginalized in textbooks and training programs, leading to a lack of evidence-based treatments, despite their clinical relevance. Our work aims to renew interest in these “forgotten” disorders.

**Objectives:**

To examine the epidemiological and clinical aspects of ATPDs through the description of clinical cases.

**Methods:**

We selected a convenience sample of 8 patients with a first diagnosis of ATPD (F23, ICD-10), currently or previously treated at Unidade Local de Saúde Amadora/Sintra. Data was collected from electronic health records, including demographic information, clinical presentation, treatment, and disease course. Additionally, we conducted a non-systematic literature review.

**Results:**

The sample consisted of 6 females and 2 males, with a median age at diagnosis of 44.5 years (min=24, max=64). Fifty percent (n=4) were migrants, with 25% (n=2) coming from lower-middle- and low-income countries. Sixty-two point five percent (n=5) received inpatient care (mean stay of 8.6 days). Most patients (87.5%, n=7) were treated with dopamine D2 receptor antagonists/partial agonists, risperidone being the most common (n=4); one patient achieved spontaneous remission. All patients had a sudden onset of symptoms; the clinical picture was primarily marked by delusions, hallucinations, confusion, psychomotor abnormalities (including catatonia signs) and mood disturbances (mainly anxiety). The mean duration of the episode was 23.2 days. All patients fully remitted and returned to their premorbid functional status. Sixty-two point five percent (n=5) experienced a second episode after a mean of 33.2 months, and 25% (n=2) had a change in diagnosis (bipolar disorder and unspecified psychosis).

**Conclusions:**

Despite the small sample and follow-up variability, our findings highlight ATPDs’ clinical heterogeneity and high recurrence rates. A renewed research effort is necessary to refine diagnostic criteria and investigate treatment responses, risk factors, and long-term prognosis of this disorder, in order to improve therapeutic strategies and patient outcomes in clinical practice.

**Disclosure of Interest:**

None Declared

